# Compliant Grasp Control Method for the Underactuated Prosthetic Hand Based on the Estimation of Grasping Force and Muscle Stiffness with sEMG

**DOI:** 10.3390/biomimetics9110658

**Published:** 2024-10-27

**Authors:** Xiaolei Xu, Hua Deng, Yi Zhang, Nianen Yi

**Affiliations:** 1 State Key Laboratory of Precision Manufacturing for Extreme Service Performance, Central South University, Changsha 410083, China; xuxiaolei@csu.edu.cn (X.X.); hdeng@csu.edu.cn (H.D.); cocacor@csu.edu.cn (N.Y.); 2 College of Mechanical & Electrical Engineering, Central South University, Changsha 410083, China

**Keywords:** prosthetic hand, grasping stiffness, surface electromyographic, fuzzy logic, compliant grasp

## Abstract

Human muscles can generate force and stiffness during contraction. When in contact with objects, human hands can achieve compliant grasping by adjusting the grasping force and the muscle stiffness based on the object’s characteristics. To realize humanoid-compliant grasping, most prosthetic hands obtain the stiffness parameter of the compliant controller according to the environmental stiffness, which may be inconsistent with the amputee’s intention. To address this issue, this paper proposes a compliant grasp control method for an underactuated prosthetic hand that can directly obtain the control signals for compliant grasping from surface electromyography (sEMG) signals. First, an estimation method of the grasping force is established based on the Huxley muscle model. Then, muscle stiffness is estimated based on the muscle contraction principle. Subsequently, a relationship between the muscle stiffness of the human hand and the stiffness parameters of the prosthetic hand controller is established based on fuzzy logic to realize compliant grasp control for the underactuated prosthetic hand. Experimental results indicate that the prosthetic hand can adjust the desired force and stiffness parameters of the impedance controller based on sEMG, achieving a quick and stable grasp as well as a slow and gentle grasp on different objects.

## 1. Introduction

When grasping an object, humans can generate grasping intentions based on the characteristics of the object and send corresponding signals to the muscles through the nervous system [1]. These signals can control the grasping of the human hand and can be observed as sEMG signals [2]. Therefore, sEMG signals can effectively reflect the grasping intentions of humans and can be used as control signals to realize the grasping action of a prosthetic hand [3]. Particularly for upper-limb amputees, myoelectric prosthetic hands can help them regain the basic operational abilities needed for daily life [4,5]. In the early stages, D. Engeberg proposed a proportional control method to estimate the magnitude of grasping force, which is also widely used in commercial prostheses [6]. At the same time, nonlinear functions can also be used to fit the relationship between grasping force and sEMG signal amplitude more accurately than the proportional relationship [7]. To further improve the accuracy of sEMG decoding, Ruyi Ma adopted the CNN classification method to estimate the grasping force levels from sEMG signals [8]. However, this method can only obtain the range of grasping force. To improve the precision of sEMG signal decoding, Hang Su used deep learning methods to estimate grasping force [9]. Due to the lack of biological models, machine learning methods are prone to overfitting and other issues [10,11]. To describe the biological characteristics of muscles, Hill and Huxley models have received widespread attention [12]. The Huxley model, a dynamic biological model, can use a kinetic model to better describe the myofilament binding reaction, which can more accurately achieve continuous estimation of grasping force, further improving the level of prosthetic hand grasping force decoding [13,14]. However, the grasping action of the human hand includes not only the grasping force but also the muscle stiffness [15]. To avoid damaging objects, the human hand often uses lower muscle stiffness when contacting objects [15]. Therefore, to achieve a compliant grasp of objects, it is necessary to further estimate the muscle stiffness during grasping on the basis of estimating the grasping force.

To better simulate the human hand and avoid damaging the grasped objects, it is necessary to control the prosthetic hand for compliant grasping based on the grasping information obtained from the sEMG signal. Compliant grasping control is mainly divided into passive compliant grasping and active compliant grasping. Passive compliance achieves compliant grasping by adding elastic elements such as springs to the prosthetic hand structure and adaptively adjusting the elastic elements when grasping objects [16]. However, directly adding elastic elements makes it difficult to adjust the grasping stiffness. Therefore, elastic elements are often accompanied by adjustment mechanisms, requiring additional structures to change parameters such as finger stiffness or damping [17]. This approach is unsuitable for prosthetic hands with high weight and volume requirements. With the development of robot control technology, active compliant control technology has attracted widespread attention because it can achieve compliant control by controlling force and position without adding mechanical structure [18]. Active compliance converts the desired compliance into the desired force and position signals, does not rely on a specific mechanical structure, and has a more flexible control method. Active compliant control is mainly divided into force–position hybrid control and impedance control. In the operation process, hybrid position–force control requires frequent switching between position control and force control due to changes in contact state, which may damage the stability of the control system [19,20]. Impedance control requires virtual impedance mass stiffness damping to enable the robot’s motion to exhibit the desired response characteristics when it is affected by the environment. The system can exhibit compliant characteristics to adapt to complex environments by adjusting the stiffness and damping parameters.

The parameter of impedance control often relies on engineering experience, and has poor adaptability when encountering unknown environments, which easily leads to large control errors and instability. To improve the stability of impedance control, adaptive impedance control with a damping adjustment strategy was proposed, and the stiffness parameter was set to zero to ensure zero force tracking error [21]. Since the stiffness parameter in the controller has a greater impact on task performance, its adjustment can easily cause instability. To address this, Vahid employed FSR contact sensors to classify the object’s stiffness and adjust the impedance parameters accordingly [22]. However, the stiffness characteristics of objects are not completely related to the fragile characteristics of objects, which may result in damage to objects. Virginia judged the required grasping stiffness based on the gestures of a prosthetic hand [23]. However, this method requires the grasping gesture and grasping stiffness to be correlated, resulting in inconvenient stiffness adjustment. Additionally, Miao learned manipulation stiffness from human demonstrations [24]. However this method depends on human demonstrations and requires relearning when the object changes. Therefore, to achieve compliant grasp control of a prosthetic hand, it is necessary to adjust the grasping force and stiffness based on a human hand’s grasping intentions. To achieve compliant grasping control of a prosthetic hand based on the grasping intention from a human hand, it is necessary to establish a connection between the stiffness of the human hand muscles and the stiffness parameters of the prosthetic hand’s controller.

In general, current research mainly focuses on estimating grasping force, while ignoring the estimation of grasping stiffness. Because of the lack of consideration of the complete human hand grasping intention, the prosthetic hand may exhibit significant stiffness parameters that result in faster convergence when grasping deformable objects, potentially causing damage. Moreover, the parameter selection in existing compliant control methods relies on engineering experience, which results in poor adaptability when in contact with the environment and may cause damage to objects due to large control errors. Most existing commercial prosthetic hands focus on obtaining grasp modes and grasp force without grasp stiffness, which is also an important parameter during contact [25]. In current research, there are machine learning-based methods for decoding stiffness from EMG signals, but machine learning has the issue of overfitting [11]. Additionally, in the compliant control of prosthetic hands, the selection of impedance parameters is often based on environmental stiffness, which may lead to inconsistency with the user’s grasp intention [18]. Therefore, obtaining the grasping force and muscle stiffness from an sEMG signal and incorporating these values in a compliant control method to comprehensively adjust the desired force and stiffness parameters of a prosthetic hand’s controller can better reflect the grasping intention of a human hand and further expand the usage scenarios of a prosthetic hand.

In this study, a novel compliant grasp control method based on the estimation of grasping force and muscle stiffness with sEMG is proposed for an underactuated prosthetic hand. The main innovations are as follows. (1) A method of estimating the grasping force and muscle stiffness of the human hand using sEMG signals is introduced based on the Huxley muscle model. The estimation results possess characteristics that indicate their suitability for application in compliance grasp control. (2) A fuzzy logic relationship between the muscle stiffness of a human hand based on sEMG and the stiffness parameters of the controller is proposed based on the characteristics of human hands grasping different objects. (3) A compliant grasping control method for the underactuated prosthetic hand is proposed to achieve the desired force and stiffness parameters obtained from sEMG. In particular, when grasping an object that is not easily damaged, greater expected force and stiffness will be applied to achieve a fast and stable grasp. On the contrary, a smaller expected force and lower stiffness parameter will be used to achieve a slow and gentle grasp, avoiding damaging the object. Experimental results show that this method can effectively adjust the desired force and stiffness parameters of the controller according to the estimated results from sEMG, thereby achieving compliant grasping of the prosthetic hand.

## 2. Problem Formulation

**Figure 1:**
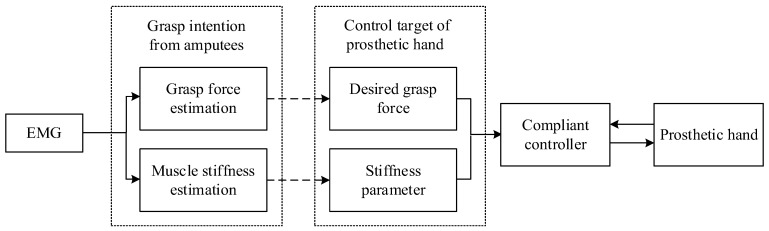
Schematic diagram of the grasping control method for the myoelectric prosthetic hand.

When grasping an object, we adjust our hand’s grasping force and muscle stiffness based on the type of object and life experience. Typically, we use lower grasping force and muscle stiffness to avoid damaging the object. To simulate this grasping method with a prosthetic hand, the controller must be able to adjust the desired force and stiffness parameters based on the grasping information estimated by sEMG when grasping different types of objects, achieving anthropomorphic compliant grasping. As shown in Figure 1, during the grasping process of the prosthetic hand, the grasping intention of the human hand needs to be converted to the objective of the prosthetic hand. When grasping the object, the controller of the prosthetic hand can use lower force and muscle stiffness to achieve compliant grasping of the object, thereby avoiding damage to it. To achieve these goals, it is necessary to estimate the grasping force and muscle stiffness of the human hand based on the sEMG signal, and then design a compliant controller based on the estimated information from sEMG to achieve compliant grasping.

## 3. Estimation of Grasping Force and Muscle Stiffness Based on Reduced-Dimension Muscle Model

To simulate the grasping method of the human hand with the prosthetic hand and achieve anthropomorphic compliant grasping control, it is first necessary to estimate the grasping force and muscle stiffness of the human hand. When grasping an object, the human brain calculates the appropriate grasping force based on the characteristics of the object and sends corresponding instructions to the muscles through the nervous system. These instructions can control the contraction of skeletal muscles and are observed in the form of sEMG signals. During the dynamic contraction of skeletal muscles, some of their properties are governed by the Huxley model. One of the main assumptions of the Huxley model is that the binding reactions of muscle filaments follow first-order kinetics, which is described by a nonlinear partial differential equation. The model parameters in the equation are related to the biological properties of muscle contraction. By solving and reducing the dimensionality of this equation, a low-order nonlinear dynamic model can be obtained, which is used for estimating the grasping force of the human hand.

According to Huxley’s skeletal muscle contraction model [12,26], the actin–myosin binding reaction obeys the kinetic equation:

(1) $$\frac{dp(x,t)}{dt}-v(t)\frac{\partial p(x,t)}{\partial x}=r(t)f(x,t)\left[1-p(x,t)\right]-g(x,t)p(x,t)$$

(2) $$r(t)={\left(e^{\gamma }-1\right)}^{-1}(e^{\gamma \alpha (t)}-1)$$

where *v*(*t*) represents the rate of myofilament slippage, *x* represents the normalized value of the distance between the cross-bridge binding site and the equilibrium position, *p*(*x*, *t*) is the distribution function of the number of cross-bridge binding sites, *f*(*x*, *t*) represents the separation rate function, *g*(*x*, *t*) represents the combination rate function, *r*(*t*) represents the activation function of the muscle, *α*(*t*) is the filtered sEMG signal, and *γ* is a constant parameter of the nonlinear shape factor.

To obtain a practical muscle model for a prosthetic hand, the following three steps are employed, as in [14]. First, the spectral method is applied to reduce the model’s dimensions. Second, the balanced truncation method is used to further reduce the dimensions and obtain an extremely low-dimensional skeletal muscle dynamic model. Finally, the time variable is integrated and a low-dimensional muscle mechanical model is obtained based on the relationship between muscle force and speed:

(3) $${\dot{\tilde{a}}}_{k}(t)={\tilde{A}}_{k}(t){\tilde{a}}_{k}(t)+{\tilde{B}}_{k}r(t),\;k=1,2,3$$

(4) $$F(t)=\sum_{k=1}^{3}C_{k}{\tilde{a}}_{k}(t)$$

where ${\tilde{a}}_{k}(t)$ is the system variable after the model’s dimensional reduction, ${\tilde{A}}_{k}(t)$ is the time-varying system coefficient after dimensional reduction, ${\tilde{B}}_{k}$ is the sEMG signal related coefficient, and $C_{k}$ is the muscle force-related coefficient.

Muscle stiffness can be defined as the change in grasping force per unit length of muscle contraction:

(5) $$K(t)=\frac{\Delta F}{\Delta X}$$

Muscle contraction speed can be approximately related to grasping force [26]:

(6) $$(F+F_{a})(v(t)+v_{b})=(F_{0}+F_{a})v_{b}$$

where $F_{0}$ is the maximum isometric contraction force of the muscle, and $F_{a}$ and $v_{b}$ are fixed coefficients.

Equation (6) can be reorganized as follows:

(7) $$v(t)=\frac{v_{b}(F_{0}+F_{a})}{F_{a}+F(t)}-v_{b}$$

Considering the muscle contraction length in time *T*:

(8) $$\Delta X=\int_{0}^{T}v(t)dt$$

the following result can be obtained by substituting (7) into (8):

(9) $$\Delta X=v_{b}\int_{0}^{T}\frac{F_{0}-F(t)}{F_{a}+F(t)}dt$$

Then, the stiffness of the muscle in this interval can be calculated as the change in grasping force per unit length of muscle contraction:

(10) $$K(t)=\frac{\Delta F}{v_{b}\int_{t-T}^{t}\frac{F_{0}-F(t)}{F_{a}+F(t)}dt}$$

The normalized stiffness parameter is defined according to the maximum measured stiffness of the muscle ($K_{\max}$) as:

(11) $$K_{a}(t)=\frac{K(t)}{K_{\max}}$$

**Figure 2:**
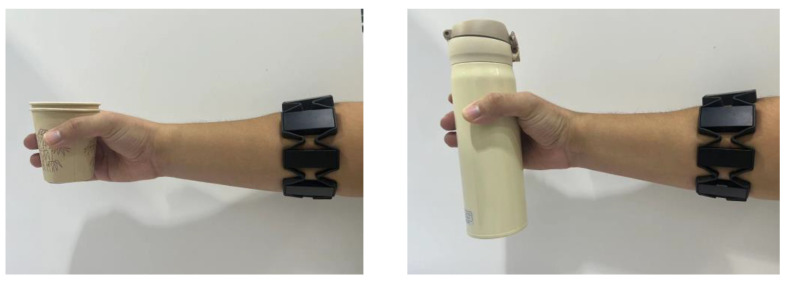
Collecting sEMG signals while grasping different objects with human hand.

**Figure 3:**
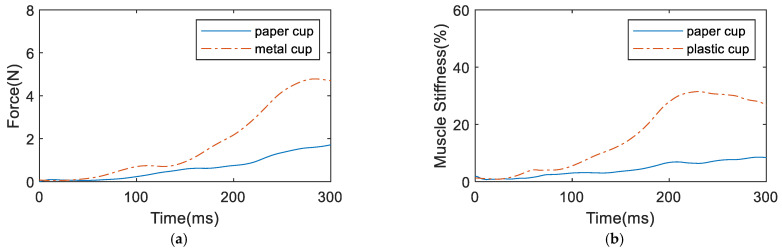
sEMG signal estimation results when grasping different objects: (**a**) grasping force, (**b**) muscle stiffness.

The identification method in [14] was used to estimate the muscle parameters. Then, a myoelectric wristband was used to collect sEMG signals and estimate the grasping force and muscle stiffness of the human hand when grasping different objects, as shown in Figure 2. The experimental results are shown in Figure 3 that for the harder and less vulnerable metal cup, the grasping force of the human hand finally reached 4.702 N and the muscle stiffness reached 26.676%. For the softer double-layer paper cup, the grasping force of the human hand finally reached 1.712 N and the muscle stiffness reached 7.617%. From the above experimental results, it can be observed that for harder objects, the grasping force and muscle stiffness of the human hand are greater and the grasping speed is faster. Conversely, for softer objects, the grasping force and muscle stiffness of the human hand are smaller and the grasping speed is slower. Therefore, there are significant differences in the grasping force and muscle stiffness estimated from sEMG when the human hand grasps different objects. These different grasping characteristics can enable the prosthetic hand to achieve compliant control for different objects.

## 4. Kinematic and Dynamic Model of the Linkage-Based Underactuated Prosthetic Hand

After obtaining the grasping force and muscle stiffness of the human hand, it is necessary to be able to control the prosthetic hand to realize the grasping intentions of the human hand. Therefore, to realize control of the prosthetic hand, it is also necessary to establish kinematic and dynamic models of the prosthetic hand.

**Figure 4:**
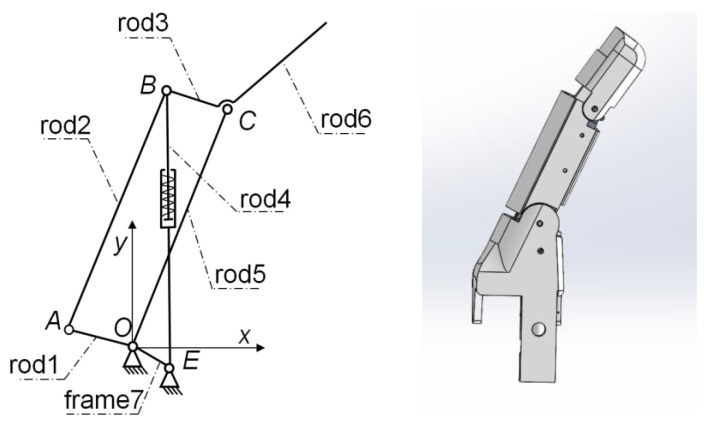
Schematic diagram of the mechanical structure of the finger on the prosthetic hand.

A linkage-based underactuated prosthetic hand was adopted in this study. Due to its flexibility and strong driving capability, a linkage-based underactuated prosthetic hand can better imitate the grasping action of the human hand. The prosthetic hand has fingers with two degrees of freedom. The coupling and adaptive grasping motions between joints are achieved through the linkage structure and passive degree-of-freedom structure. Its finger consists of six rods, as shown in Figure 4. Among them, rod 4 has an extension degree of freedom. When the proximal end contacts the object and the clamping force is greater than the preload of rod 4, rod 4 will extend and deform. At this time, the movement of the thumb will change from the coupled motion of rods 5 and 6 to adaptive motion.

When the motor drives pull rod 1 through the reducer, a closed-chain structure is formed. The proximal phalanx rod (5) and the distal phalanx rod (6) of the thumb produce coupled motion. Assume that the geometric center coordinates of rod n are $\left(x_{n},y_{n}\right)$, the length is $l_{n}$, the x-axis is used as a reference, and the rotation angle is $\theta_{n}$. According to the geometric relationship [27], the Lagrangian method is used to establish the dynamic equation of the thumb hand:

(12) $$L=\sum_{n=1}^{5}\left[\frac{1}{2}I_{n}{\dot{\theta }}_{n}^{2}+\frac{1}{2}m_{n}({\dot{\tilde{x}}}_{n}^{2}+{\dot{\tilde{y}}}_{n}^{2})-m_{n}g{\tilde{y}}_{n}\right]$$

(13) $$\frac{d}{dt}\left(\frac{\partial L}{\partial {\dot{\theta }}_{1}}\right)-\frac{\partial L}{\partial \theta_{1}}=\tau_{1}-J^{-1}F-f$$

where $I_{n}$ represents the moment of inertia of the nth connecting rod, $m_{n}$ represents the mass of the nth connecting rod, $\left({\tilde{x}}_{n},{\tilde{y}}_{n}\right)$ represents the center-of-mass coordinate of the nth connecting rod, g represents the acceleration of gravity, $\tau_{1}$ represents the driving force of connecting rod 1, F represents the grasping force, and f represents the friction force.

The relationship between the geometric center and the centroid can be obtained:

(14) $${\tilde{x}}_{n}+i{\tilde{y}}_{n}=x_{n}+iy_{n}+{\tilde{l}}_{n}e^{i(\theta_{n}+{\tilde{\theta }}_{n})},n=1,2,3,4,5$$

where ${\tilde{l}}_{n}$ represents the distance offset of connecting rod *n* and ${\tilde{\theta }}_{n}$ represents the rotation offset of the connecting rod *n*.

When the prosthetic fingertip contacts an object, let the contact point be *p*. At this time, the contact point equation is:

(15) $$x_{p}+iy_{p}=x_{3}+iy_{3}+0.5l_{3}e^{i\theta_{3}}+l_{p}e^{i\theta_{p}}$$

After combining (14) with the contact point Equation (15), the Jacobian matrix can be obtained according to the velocity relationship:

(16) $${\left[{\dot{x}}_{p},{\dot{y}}_{p}\right]}^{T}=J{\dot{\theta }}_{1}$$

Finally, the fingertip contact dynamic model of the prosthetic hand can be obtained as follows:

(17) $$M{\ddot{\theta }}_{1}+C{\dot{\theta }}_{1}^{2}+G=\tau_{1}-J^{T}F-f$$

where M is the mass matrix, C is the velocity-related term, and G is the gravity matrix.

## 5. The Compliant Grasp Control Method for the Underactuated Prosthetic Hand

**Figure 5:**
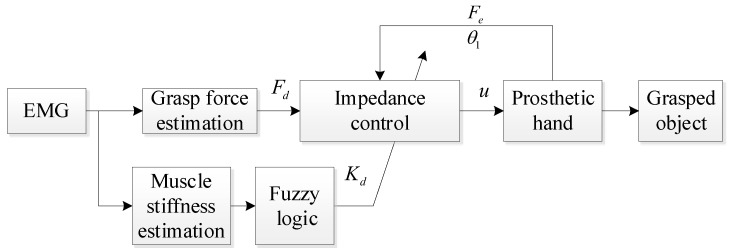
Schematic of the compliant control algorithm based on grasping force and muscle stiffness estimation.

After obtaining the dynamic model of the underactuated prosthetic hand, a compliant grasping control algorithm should be designed based on the grasping information from the sEMG signal. The specific control method is as follows. First, the grasping action signal is generated by decoding the operator’s sEMG signal. Before the prosthetic hand touches the object, the motor will be adjusted to a lower value to avoid damage when contacting the object. When the prosthetic hand contacts the object, the prosthetic hand will adjust the convergence target and stiffness parameters of the controller according to the grasping force and muscle stiffness of the human hand obtained by the sEMG signal, thereby achieving a compliant grasp of the object, as shown in Figure 5.

### 5.1. The Compliant Grasp Control Method Based on Muscle Stiffness

To achieve compliant grasping, the magnitude of the grasping force and the convergence rate of the force should be adjusted according to the characteristics of the grasped object. To avoid damaging objects, the grasping force of the prosthetic hand should be lower and applied more slowly, achieving slow and gentle grasping. For objects that are less prone to damage, the desired grasping force can be greater and converge quickly to achieve stable grasping.

According to (17) the prosthetic hand model can be organized as follows:

(18) $${\ddot{\theta }}_{1}=M{\left(\theta_{1}\right)}^{-1}\left[\tau_{1}-J^{-1}F-f({\dot{\theta }}_{1})-C(\theta_{1}){\dot{\theta }}_{1}^{2}-G(\theta_{1})\right]$$

The error is defined by the desired position $\theta_{1d}$ as follows:

(19) $$e=\theta_{1}-\theta_{1d}$$

To simulate the muscle stiffness of human hands, the following controller can be designed based on impedance control:

(20) $$u=M(\theta_{1})\Gamma_{1}(e)+J^{-1}F+\tilde{f}({\dot{\theta }}_{1})+C(\theta_{1}){\dot{\theta }}_{1}^{2}+G(\theta_{1})$$

(21) $$\Gamma_{1}(e)={\ddot{\theta }}_{1d}-M_{d}^{-1}[B_{d}\dot{e}+K_{d}e+(F-F_{d})]$$

where $M_{d}$,$B_{d}$,$K_{d}$,${\ddot{\theta }}_{1d}$ and $F_{d}$, are the desired inertia, damping, stiffness, acceleration, and grasp force, respectively.

**Figure 6:**
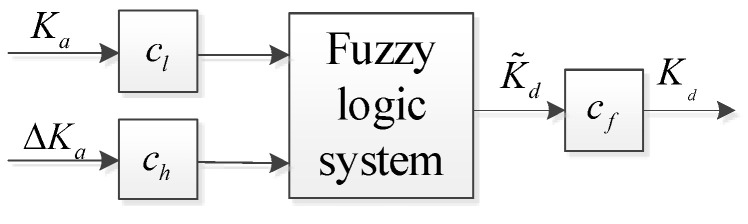
The fuzzy logic relationship between estimated muscle stiffness and controller stiffness.

When the impedance parameters are set to values similar to those of the human hand, a natural control feel similar to that of the original limb can be achieved [28]. To achieve an anthropomorphic muscle stiffness adjustment strategy, the following fuzzy rules in Table 1 and Figure 6 are used to adjust the stiffness of the object. The standard triangular membership functions are used as the input and output membership functions. The fuzzy rule was used as follows: if $K_{a}$ is $M_{i}$ and $\Delta K_{a}$ is $N_{i}$, then ${\tilde{K}}_{d}$ is $G_{i+j}$, where $M_{i}$ and $N_{i}$ denote the input fuzzy sets and $G_{i+j}$ denotes the output fuzzy set. The details of the rules are shown in Table 1, where $K_{a}$ and $\Delta K_{a}$ are defined as five fuzzy sets: very small (VS), small (SL), medium (ME), relatively large (RL), and large (LE). The output of the fuzzy estimation is the stiffness parameter ${\tilde{K}}_{d}$, which is also defined as five fuzzy sets: very small (VS), small (SL), medium (ME), relatively large (RL), and large (LE). The fuzzy logic relationship can adjust the controller’s stiffness parameters based on the muscle stiffness of the human hand. When the muscle stiffness and its variation are high, the controller’s stiffness parameters are set higher to ensure a quick and stable grasp of the prosthetic hand on the object. Conversely, when the muscle stiffness and its variation are low, the controller’s stiffness parameters are set lower, allowing the prosthetic hand to perform a compliant grasp when in contact with the object.

### 5.2. Stability of the Proposed Compliant Grasp Control Method

Substituting into the model, we can obtain the following impedance model:

(22) $$M_{d}\ddot{e}+B_{d}\dot{e}+K_{d}e=-(F-F_{d})$$

When the contact force is calculated by the object stiffness:

(23) $$M_{d}\ddot{e}+B_{d}\dot{e}+K_{d}e=-KJ^{-1}e$$

(23) can be reorganized as follows:

(24) $$\ddot{e}+B_{d}M_{d}^{-1}\dot{e}+(K_{d}+KJ^{-1})M_{d}^{-1}e=0$$

Considering the following Lyapunov function:

(25) $$V=\frac{1}{2}{\dot{e}}^{T}\dot{e}+\frac{1}{2}e^{T}(K_{d}+KJ^{-1})M_{d}^{-1}e$$

after derivation:

(26) $$\dot{V}={\dot{e}}^{T}\ddot{e}+e^{T}(K_{d}+KJ^{-1})M_{d}^{-1}\dot{e}$$

Substitute (24) into (26), and the following result can be obtained:

(27) $$\dot{V}=-{\dot{e}}^{T}B_{d}M_{d}^{-1}\dot{e}$$

When $(K_{d}+KJ^{-1})M_{d}^{-1}>0$ and $B_{d}M_{d}^{-1}>0$:

(28) $$\dot{V}\leq 0\mathrm{and}\;V\geq 0$$

As a result, Lyapunov stability conditions are satisfied.

## 6. Experiment on Compliant Grasp Control of Underactuated Prosthetic Hand

To verify the compliant grasping force control method of the underactuated myoelectric prosthetic hand proposed in this paper, grasping experiments of different objects were designed for verification. During the experiment, the prosthetic hand was used to grasp four objects: a single-layer paper cup, double-layer paper cup, milk carton, and plastic cup. Among them, the single-layer paper cup and double-layer paper cup are prone to excessive deformation during grasping, resulting in irreversible damage to the grasped objects. A differently shaped milk carton and plastic cup were also used as the comparison group and were not easily damaged during the grasping process. The grasping experiments on the above four objects were used to verify the grasping ability of the compliant grasp control method proposed in this paper.

The instruments used in the experiments were as follows. The prosthetic hand employs Faulhaber 1028B series brushless servo motors from Germany. The reducer is a Faulhaber planetary gear reducer with a reduction ratio of 1024:1. The motors are controlled by MCDC3-3002 drivers from Faulhaber with the CAN protocol. The whole system is controlled by an stm32H750 microcontroller with a main frequency of 480 MHz. The experimental data are sent to a host computer with LabVIEW for display and recording through a serial port. The control and signal feedback frequency of the microcontroller is 1 kHz. A FlexiForce force sensor with an optional range of 4.4 N is installed on the finger to measure the contact force. Polynomial fitting is used to ensure measurement accuracy, with a measurement error of less than 3% of full scale. The EMG signals are collected using a MYO armband from Thalmic Labs, Canada. This armband features eight channels for EMG signals and can transmit data via Bluetooth, with an overall sampling frequency of 200 Hz. When wearing the armband, the arm is positioned horizontally in front of the chest, with the armband placed approximately 10 cm from the elbow joint. Then, the armband is rotated until the first channel is on the outer side of the arm and parallel to the body.

**Figure 7:**
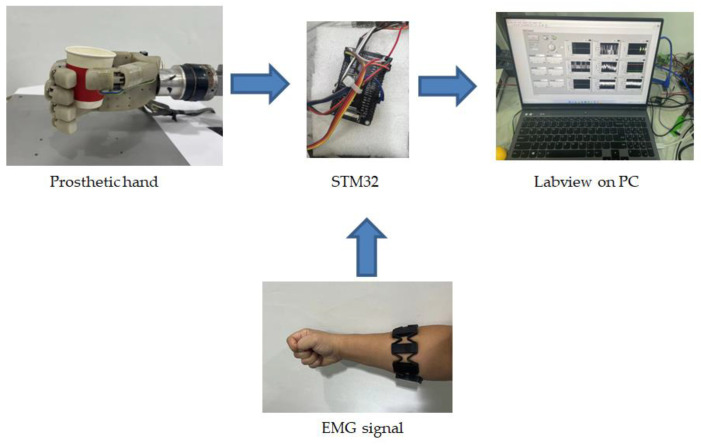
Schematic diagram of the experimental process of the grasping force and muscle stiffness estimation-based compliant grasp control of the underactuated prosthetic hand.

**Figure 8:**
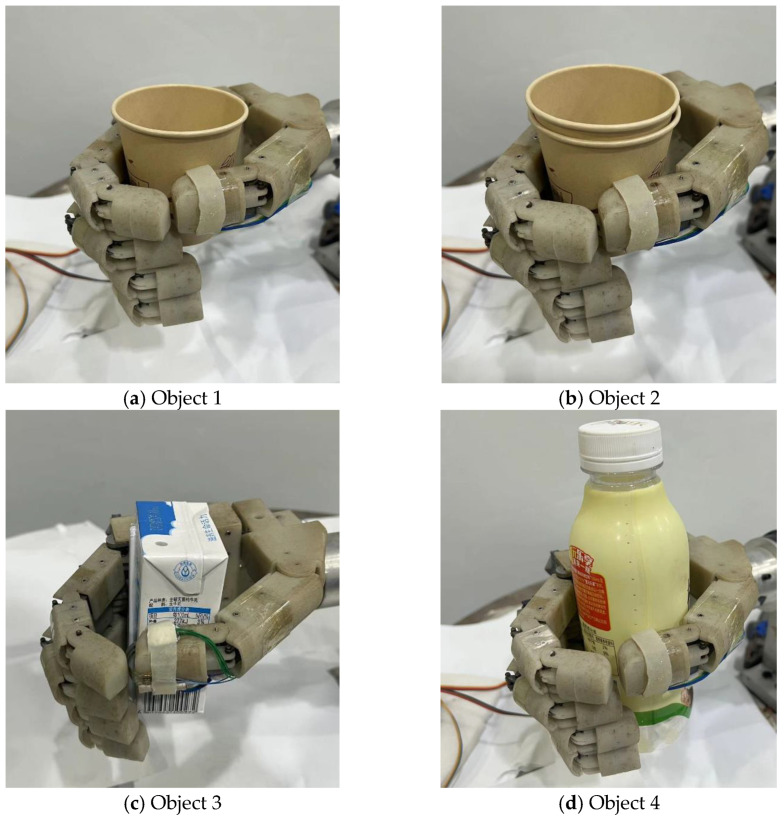
Experiments of the compliant grasp control with single-layer paper cup (Object 1), double-layer paper cup (Object 2), milk carton (Object 3), and plastic cup (Object 4).

The grasping control experiment process is shown in Figure 7. The operator maintains a fist gesture to simulate the sEMG signal of amputees when grasping. The prosthetic hand will enter the compliant grasping control mode when it detects contact with the object. At this time, the control algorithm will adjust the desired grasping force according to the grasping force decoded by the sEMG signal. Additionally, the stiffness parameter of the controller is adjusted by the muscle stiffness estimated from sEMG signals according to fuzzy logic relationships to achieve the grasping effect expected by the operator. As illustrated in Figure 8, the experimental process involves controlling the prosthetic hand to grasp the single-layer soft paper cup, a double-layer paper cup, a milk carton, and a plastic cup. During this process, the input signals, including the estimated grasping force and grasping stiffness from sEMG, as well as the control signals of the prosthetic hand, such as driving voltage, driving current, rotation angle, and grasping force, are recorded.

**Figure 9:**
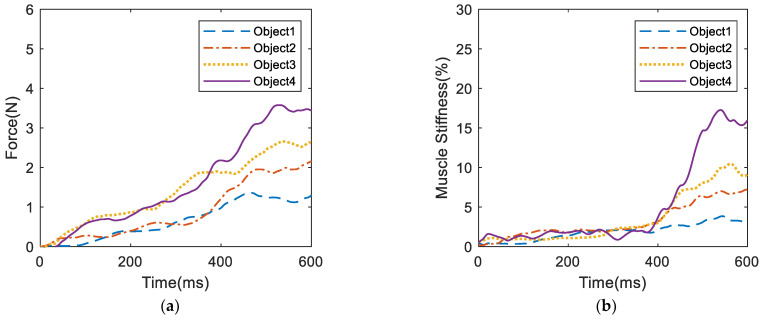
Experimental results of sEMG signal estimation when grasping different objects: (**a**) estimated grasping force, (**b**) estimated muscle stiffness.

**Figure 10:**
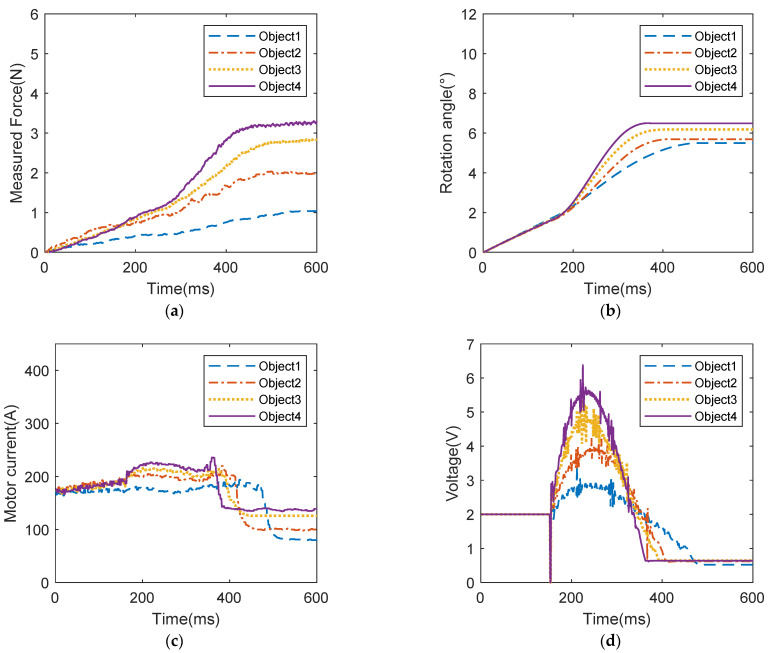
Experimental results of the compliant grasp control experiment: (**a**) grasping force applied by the prosthetic hand, (**b**) rotation angle of rod 1, (**c**) motor current, (**d**) motor voltage.

The experimental results of the first group are shown by the dotted lines in Figure 9 and Figure 10. When grasping a single-layer paper cup (Object 1), the grasping force estimated from sEMG is the smallest compared with the other three objects—1.298 N. At this time, the estimated muscle stiffness is 3.301%, which is the smallest compared with the other three objects. The stiffness parameter of the controller estimated using fuzzy logic reaches 22.89. This shows that when the human hand grasps the object, to avoid damage to Object 1, a smaller grasping force and muscle stiffness are used. At this time, it can be seen from Figure 10a,b that the final grasping force applied reaches 1.978 N, and the maximum rotation angle of the motor is 5.685°. From Figure 10c,d, it can be seen that the grasping speed of the prosthetic hand is also the slowest under the smaller expected force and stiffness parameters, and the grasping action is not completed until 481 ms. These results indicate that the prosthetic hand uses less grasping force and grasping speed to ensure a natural and gentle grasping action, thereby minimizing the risk of damaging Object 1.

The results of the second group are shown in the dotted lines in Figure 9 and Figure 10. When grasping the double-layer paper cup (Object 2), the grasping force decoded by myoelectric decoding is medium, reaching 2.160 N, and the muscle stiffness decoded by myoelectric decoding is 7.191%. The stiffness parameter of the controller estimated using fuzzy logic reached 54.41. This shows that since the object is less susceptible to damage than Object 1, the human hand will use medium grasping force and muscle stiffness to grasp Object 2. At this time, it can be seen from Figure 10a,b that the final grasping force applied reached 1.034 N, and the maximum rotation angle of the motor was 5.499°. It can be seen from Figure 10c,d that the grasping speed of the prosthetic hand is also moderate under the medium expected force and stiffness parameter, and the grasping action is completed at 408 ms. This demonstrates that the prosthetic hand uses medium expected force and grasping speed to grasp Object 2. What is more, these results highlight the prosthetic hand’s capability to adjust its control parameters based on the decoded muscle stiffness and grasping force of the human hand, ensuring a balanced and efficient grasping performance that aligns with the varying requirements of different objects.

The experimental results of the third group are shown as dotted lines in Figure 9 and Figure 10. When grasping the milk carton (Object 3), the electromyography (EMG)-decoded grasping force is greater than that for the double-layer paper cup, reaching 2.632 N. At this time, the muscle stiffness decoded by EMG is 9.137%. The stiffness parameter of the controller estimated using fuzzy logic reached 63.60. This indicates that the human hand exerts greater grasping force and stiffness when grasping this object compared to the double-layer paper cup, although the difference is not significant. From Figure 10a,b, it can be seen that the final applied grasping force reached 2.570 N and the maximum rotation angle of the motor was 6.178°. From Figure 10c,d, it can be observed that the increased grasping stiffness results in a faster grasping speed. Therefore, the final convergence time is shorter than the third group, at 393 ms. This demonstrates that the prosthetic hand not only has the greater grasping force but also the faster grasping speed, and can quickly and stably grasp objects according to the intention of the human hand when grasping Object 3.

The experimental results of the fourth group are shown in the solid lines in Figure 9 and Figure 10. When grasping the plastic cup (Object 4), the grasping force estimated from sEMG is the greatest compared with the other three objects, reaching 3.437 N. At this time, the estimated muscle stiffness is also high, reaching 15.954%. The stiffness parameter of the controller estimated using fuzzy logic reached 72.69. This shows that the human hand adopts greater grasping force and muscle stiffness when it grasps Object 4 without concern about the object being damaged. At this time, it can be seen in Figure 10a,b that the final grasping force applied by the prosthetic hand reaches 3.278 N and the maximum rotation angle of the motor is 6.493°. In Figure 10c,d, it can be observed that with greater desired force and stiffness, the prosthetic hand also achieved the fastest grasping speed, completing the grasping action in 367 ms. This demonstrates that the prosthetic hand not only has the greatest grasping force but also the fastest grasping speed based on the intention of the human hand when grasping Object 4.

Four groups of experiments showed that when grasping an object, the human hand can adjust the grasping force and muscle stiffness according to the characteristics of the object. By setting the stiffness parameters of the controller to values similar to those of the human hand, the desired force and stiffness parameters of the prosthetic hand can be controlled based on the person’s judgment of the object’s characteristics to achieve compliant grasping of different objects.

## 7. Discussion

To simulate the grasping characteristics of the human hand, compliant control methods can be used for prosthetic hand control. However, current compliant control methods for prosthetic hands often require the prosthetic hand to sense the characteristics of the object to obtain the controller’s stiffness parameters, which not only necessitates additional sensors but also may be inconsistent with the amputee’s intention. To address this issue, this paper proposes a compliant grasping control method for an underactuated prosthetic hand based on the estimation of grasping force and muscle stiffness with sEMG. This method adjusts the prosthetic hand controller based on the grasping force and muscle stiffness estimated from sEMG signals, thereby avoiding damage to the object. During the experiments, the milk carton (Object 3) and the plastic cup (Object 4) exhibited the greater stiffness, making them less prone to deformation and damage. Therefore, when grasping the milk carton (Object 3) and the plastic cup (Object 4), the human hand used greater grasping force and muscle stiffness. In contrast, the single-layer paper cup (Object 1) and the double-layer paper cup (Object 2) were more susceptible to over-deformation and damage, prompting the human hand to use relatively lower grasping force and muscle stiffness. This operational strategy of the human hand is reflected in the prosthetic hand through sEMG signals. When grasping the milk carton (Object 3) and the plastic cup (Object 4), the prosthetic hand used greater desired force and stiffness, achieving a quick and stable grasp. For the single-layer paper cup (Object 1) and the double-layer paper cup (Object 2), the prosthetic hand adopted less desired force and stiffness, resulting in a slow and gentle grasp that avoided damaging the objects.

To implement the proposed method, the force sensor and microcontroller can be added to existing prosthetic hands without replacing the bio-interface, thereby saving costs and ensuring comfort usage. Moreover, due to the good interactivity of STM32, there is broad potential for large-scale implementation. Therefore, this method holds promising research and application prospects for prosthetic hand grasping. It can achieve human-like grasping from the sEMG signal, providing a new control method for grasping force in low-cost prosthetic hands. In addition, the proposed method uses a low-power microcontroller with limited computational load, which can be easily integrated into commercial prosthetic hands, offering promising application prospects.

## 8. Conclusions

In summary, based on the characteristics of a multi-link underactuated prosthetic hand, this study proposed a compliant grasping control method for an underactuated prosthetic hand based on the estimation of grasping force and muscle stiffness with sEMG. This method can estimate the grasping force and muscle stiffness of the human hand using collected sEMG signals. By further integrating impedance grasping control algorithms, the prosthetic hand’s controller adjusts the desired force and stiffness parameters to prevent damage to the object during grasping. Since this method is based on human grasping, it may result in failures when attempting to grasp unfamiliar objects, requiring multiple attempts and learning. In the future, we aim to further enhance the grasping ability and expand the application scenarios of the prosthetic hand by combining grasping and perception capabilities similar to the human hand.

## Figures and Tables

**Table 1:** Fuzzy logic relationship of the compliant controller.

${\tilde{K}}_{d}$		$\Delta K_{a}$				
		LE	RL	ME	SL	VS
$K_{a}$	LE	LE	LE	RL	ME	SL
	RL	LE	RL	ME	ME	SL
	ME	LE	RL	ME	SL	SL
	SL	RL	ME	ME	VS	VS
	VS	RL	ME	SL	VS	VS

## Data Availability

The data and code of the current study can be obtained from the corresponding author upon reasonable request.
